# Accurate Paediatric Brain Tumour Classification Through Improved Quantitative Analysis of ^1^H MR Imaging and Spectroscopy

**DOI:** 10.1002/nbm.70103

**Published:** 2025-07-23

**Authors:** Teddy Zhao, Heather E. L. Rose, James T. Grist, Lesley MacPherson, Huijun Li, Theodoros N. Arvanitis, John R. Apps, Andrew C. Peet

**Affiliations:** ^1^ Cancer and Genomic Sciences University of Birmingham Birmingham UK; ^2^ Oncology Birmingham Children's Hospital Birmingham UK; ^3^ Oxford Centre for Clinical Magnetic Resonance Research University of Oxford Oxford UK; ^4^ Radiology University of Oxford Oxford UK; ^5^ Radiology Birmingham Children's Hospital Birmingham UK; ^6^ Engineering University of Birmingham Birmingham UK

**Keywords:** diffusion‐weighted magnetic resonance imaging, machine learning, magnetic resonance spectroscopy, multimodal imaging, paediatric brain tumour

## Abstract

Multimodality imaging is an emerging research topic in neuro‐oncology for its potential of being able to demonstrate tumours in a more comprehensive manner. Diffusion‐weighted magnetic resonance imaging (dMRI) and proton magnetic resonance spectroscopy (^1^H‐MRS) allow inferring tissue cellularity and biochemical properties, respectively. Combining dMRI and ^1^H‐MRS may provide more accurate diagnosis for paediatric brain tumours than only one modality. This retrospective study collected 1.5‐T clinical ^1^H‐MRS and dMRI from 32 patients to assess paediatric brain tumour classification with combined dMRI and ^1^H‐MRS. Specifically, spectral noise of ^1^H‐MRS was suppressed before calculating metabolite concentrations. Extracted radiomic features were apparent diffusion coefficient (ADC) histogram features through dMRI and metabolite concentrations through ^1^H‐MRS. These features were put together and then ranked according to the multiclass area under the curve (mAUC) and selected for tumour classification through machine learning. Tumours were precisely typed by combining noise‐suppressed ^1^H‐MRS and dMRI, and the cross‐validated accuracy was improved to be 100% according to naïve Bayes. The finally selected radiomic biomarkers, which showed the highest diagnostic ability, were ADC fifth percentile (mAUC = 0.970), *myo*‐inositol (mAUC = 0.952), combined glutamate and glutamine (mAUC = 0.853), total creatine (mAUC = 0.837) and glycine (mAUC = 0.815). The study indicates combining MR imaging and spectroscopy can provide better diagnostic performance than single‐modal imaging.

## Graphical abstract

This study presents a method of quantitative analysis in 1.5‐T short‐*T*_E_^1^H‐MRS and dMRI for paediatric brain tumour classification. Accurate diagnostic accuracy of ependymomas, medulloblastomas and pilocytic astrocytomas can be achieved through combining the two modalities, which is significantly better than only one modality or without ^1^H‐MRS noise suppression.

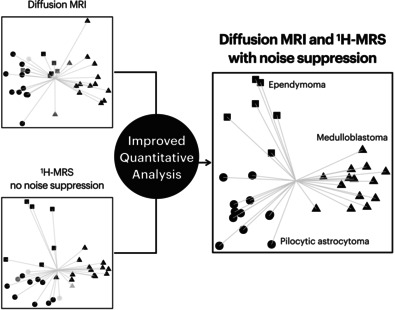

## 1 Introduction

Paediatric brain tumours are among the most common solid cancers in children and often have a poor outcome [1]. Early diagnosis of paediatric brain tumours can improve long‐term outcome [2], in which MRI has been used as the key modality for cancer early detection and diagnosis [3] in the current clinical practice [4]. Conventional MRI provides surgical references yet limited diagnostic performance, while advanced modalities can identify some key biomarkers that have added diagnostic value for classifying paediatric brain tumours [5]. Proton MR spectroscopy (^1^H‐MRS) [5] allows for the non‐invasive assessment of in vivo metabolite profiles that can be used to probe the underlying biology of tumour progression [7] and treatment response [8]. Diffusion‐weighted MRI (dMRI) [9] can demonstrate brain tumour cellularity by measuring microscopic water diffusion that inversely correlates with cell density [10]. Both modalities provide quantitative radiomic biomarkers that show their benefit in early diagnosis and clinical management of brain tumours [11]. Along with biomarker discovery studies, computational analysis attracts attention for its potential of improving and interpreting the biomarkers [12, 13]. For improving data quality purposes, noise‐suppressed dMRI and ^1^H‐MRS may detect or type tumours more sensitively [14, 15]. Computation also addresses data harmonisation [16] and interpretation [17] for multicentre or multiprotocol datasets. Such work is considered to have the potential of being translated into clinical practice to improve early diagnosis and patient outcome [18].

^1^H‐MRS or dMRI have been investigated for their diagnostic value of paediatric brain tumours individually (Table 1). The metabolite concentrations observed through clinical ^1^H‐MRS are considered as promising biomarkers for identifying brain tumours, and they were found to allow higher diagnostic performance over conventional imaging [19]. In comparison to classic pipelines, spectroscopy analysis, like combining multiple *T*_E_ [20], metabolite selection [17] or spectral noise suppression [15], was reported to be able to further improve diagnostic performance. Meanwhile, dMRI can provide apparent diffusion coefficients (ADCs) as imaging biomarkers that characterise tissue cellularity [9]. There have been shown to be differences in ADCs between common paediatric brain tumour types [21, 22]. For example, ADC features were considered significant for differentiating medulloblastomas and pilocytic astrocytomas, especially the minimum ADC [23, 24]. Metabolite profiles and ADC histogram metrics can achieve approximately 92% and 85% accuracy for commonly occurring paediatric brain tumours on multicentre bases [15, 25]. These findings indicate the potential of the combination of ^1^H‐MRS and dMRI for further improving paediatric brain tumour classification.

This study presents a method of processing and selecting features from 1.5‐T short‐*T*_E_^1^H‐MRS and dMRI and assesses their combined diagnostic accuracy for paediatric brain tumours.

**TABLE 1:** Summary of the research progress for paediatric posterior fossa tumour diagnosis with dMRI and/or ^1^H‐MRS.

Authors	Year	Tumour types				Features	Balanced accuracy
		EP	MB	PA	Total		EP|MB|PA
dMRI							
Yamasaki et al. [26]	2005	6	/	23^a^	29	ADCs	Not reported
Rumboldt et al. [27]	2006	5	8	/	13	ADCs	Not reported
Yamashita et al. [24]	2009	4	11	3	18	ADC histogram features	Not reported
Bull et al. [28]	2012	5	16	9^b^	22	ADC histogram features	91%
R. Gutierrez et al. [21]	2014	7	17	16	40	ADC histogram features	96%
Novak et al. [25]	2021	26	55	36	117	ADC histogram features	84%–86%
^1^H‐MRS							
Davies et al. [29]	2008	5	18	12	35	Metabolite profile	94%
Raschke et al. [30]	2012	5	18	13	36	Spectrum	88%
Vicente et al. [20]	2013	11	29	38	78	Metabolite profile	67%–94%
Zarinabad et al. [31]	2017	10	38	42	90	Metabolite profile	91%
						Spectrum	81%
Zarinabad et al. [32]	2018	4	17	20	41	Metabolite profile	86%
Manias et al. [33]	2018	4	14	13	31	Metabolite profile	64%–91%
Davies et al. [29]	2022	4	18	12	34	Metabolite profile	91%
Zhao et al. (Cohort 1) [17]	2022	13	31	39	83	Metabolite profile	72%–80%
Zhao et al. (Cohort 2) [17]	2022	4	17	21	42	Metabolite profile	46%–53%
Zhao et al. (Cohort 1) [15]	2024	13	31	39	83	Metabolite profile	84%–90%
Zhao et al. (Cohort 2) [15]	2024	4	17	21	42	Metabolite profile	52%–61%
dMRI and ^1^H‐MRS							
Schneider et al. [34]	2007	1	7	4	12	ADCs and metabolite profiles	Not reported

*Note:* Table showing the research progress of paediatric posterior fossa tumour classification through diffusion‐weighted MRI and/or proton MR spectroscopy raw data or extracted biomarkers. Only the three tumour types, namely, ependymomas, medulloblastomas and pilocytic astrocytomas are included in this table, and other tumour types have been omitted if included in those studies. The classification accuracy is given through either machine learning or basic statistical analysis whichever presented, and the results are presented as a range if more than one classifier are discussed. Each row of the table presents the results of only one cohort, and papers that include more than one cohort have been split into multiple lines. The details of physical parameters and computational settings, such as field strength and feature extraction methods, are presented in the Supporting Information.

Abbreviations: ^1^H‐MRS, proton magnetic resonance spectroscopy; ADC, apparent diffusion coefficient; dMRI, diffusion‐weighted magnetic resonance imaging; EP, ependymoma; MB, medulloblastoma; PA, pilocytic astrocytoma.

^a^ Including 6 pilocytic astrocytomas and 17 juvenile pilocytic astrocytomas.

^b^ All are juvenile pilocytic astrocytomas.

**TABLE 2:** Acquisition details for diffusion magnetic resonance imaging (dMRI) and proton magnetic resonance spectroscopy (^1^H‐MRS).

Parameter	Value
Both dMRI and ^1^H‐MRS	
Field strength (T)	1.5
Manufacturers	Siemens Healthcare (Erlangen, Germany);
	General Electric Medical Systems (Milwaukee, WI, USA)
Models	Siemens Symphony MAGNETOM, Avanto, Area; GE Signa LX
RF coil	^1^H head coil
dMRI only	
Acquisition matrix	128×128–384×384
Bandwidth (Hz)	870–1953.12
b value (s/mm^2^)	0 and 1000
Echo time (ms)	84.3–161
Field of view (mm^2^)	162.5×200
Number of slices	19–48
Repetition time	5300–9300
Sequence	Echo planar imaging (EPI)
Slice gap (mm)	4–6.5
Slice thickness (mm)	4–5
^1^H‐MRS only	
Chemical shift displacement per ppm	< 4%
Echo time (ms)	30
Number of averages	128
Number of complex points	512 or 2048
Number of channels in head coils or head and neck coils	8–32
Repetition time (ms)	1500–2000
Sampling frequency (Hz)	2000–2500
Sequence	Single‐voxel point‐resolved spectroscopy sequence (PRESS)
Total number of acquisitions per spectrum	128 or 256
Volume of interest size (mm^3^), nominal	15×15×15–20×20×20
Water suppression	Chemical shift selective saturation pulses, no out of volume suppression

## 2 Materials and Methods

### 2.1 Patients

Patients were recruited between October 2004 and December 2019 in Birmingham Children's Hospital, Birmingham, England to the *Functional Imaging of Tumours CNS 2004/10* study. This study was approved by the research ethics committee (ethics number: 04/MRE04/41). Informed consent was obtained from parents or guardians of patients. All patients underwent structural MRI, ^1^H‐MRS and dMRI examination before receiving surgical resection. Patients diagnosed to have a posterior fossa tumour, ependymoma, medulloblastoma or pilocytic astrocytoma were included in this study, where diagnosis was confirmed with histology and reviewed at local tumour boards.

**FIGURE 1:**
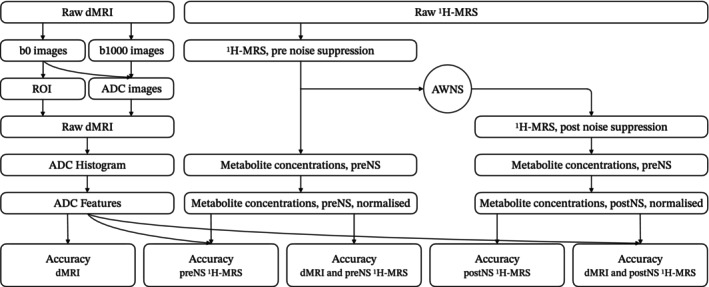
Flowchart of data analysis in the study. Abbreviations: ^1^H‐MRS, proton magnetic resonance spectroscopy; Acc., accuracy; ADC, apparent diffusion coefficient; AWNS, adaptive wavelet noise suppression; dMRI, diffusion‐weighted magnetic resonance imaging; postNS, postnoise suppression; preNS, prenoise suppression.

### 2.2 Data Acquisition

Patient data were acquired on four 1.5‐T scanners (Table 2) [35]. Structural MRI included *T*_1_‐weighted, *T*_2_‐weighted and *T*_1_‐weighted postcontrast sequences. ^1^H‐MRS with water reference acquisition was performed after conventional imaging that included gadolinium administration. Volumes of interest (VOIs) were manually placed to be completely within the solid regions of tumours according to structural images, with contrast enhancement and low ADC being used as guides where tumours exhibited some heterogeneity.

### 2.3 dMRI Analysis

The regions of interest (ROIs) of brain tumours that were exclusive of cysts for dMRI were defined on b0 images by a radiologist (H.L.) with more than 10 years of experiences [36]. The ROIs were visually built by linking the defined vertices and reviewed by an oncologist (A.C.P.) who had more than 30 years of experiences, where cases were screened based on data quality and availability (Table 3). All these steps were performed by using OsiriX (Version 12.0, Pixmeo SARL, Geneva, Switzerland).

**TABLE 3:** Patient screening according to data quality.

	EP	MB	PA	Total
Initial	16	38	54	108
Excluded				
^1^H‐MRS				
Modality unavailable	2	4	3	9
Partial volume effects	1	1	3	5
Poor FWHM	1	0	1	2
Poor fSNR	2	0	2	4
Artefacts	1	2	6	9
dMRI				
Modality unavailable	3	18	25	46
b1000 images unavailable	0	1	0	1
Final	6	12	14	32

*Note:* Table showing the details of patient exclusion based on the data quality of diffusion‐weighted MRI and proton MR spectroscopy.

Abbreviations: ^1^H‐MRS, proton magnetic resonance spectroscopy; dMRI, diffusion‐weighted magnetic resonance imaging; EP, ependymoma; fSNR, fitting‐based signal‐to‐noise ratio; FWHM, full‐width at half maximum; MB, medulloblastoma; PA, pilocytic astrocytoma.

The VOIs were reconstructed by combining the ROIs in each of the related b0 slices, by using in house software. These VOIs were then applied to ADC images, which were generated from the MR scanners, to derive ADC histograms and extracting ADC features. Extracted ADC features include percentiles from the 5th to the 95th, mean, median, variance, maximum, minimum, skewness, mode, kurtosis and entropy. ADC image analysis (Figure 1), including VOI reconstruction, was performed on MATLAB (Version 2021a, MathWorks, Natick, MA, USA).

### 2.4 Spectroscopy Processing and Quality Control

^1^H‐MRS was quantified by using TARQUIN (Version 4.3.11) with ^1^H brain full basis set that includes lipid and macromolecule signals [37]. Quantification analysis includes phasing, chemical shift calibration, eddy current correction and metabolite amplitude estimation. In this process, the baseline is modelled by using a spline function and does not include lipids and macromolecules. Lipid and macromolecule signals are modelled by using the peak functions in the quantification software and included in the analysis output. Zero‐filling is performed to 2048 points, and no initial data points are specifically removed [35]. ^1^H‐MRS was then processed with adaptive wavelet noise suppression for improving spectral quality [15]. The output measure for spectral analysis is absolute concentration by using water as the reference [35, 37]. For assessing the quality of ^1^H‐MRS, the fitting‐based signal‐to‐noise ratio (fSNR) and full‐width at half maximum (FWHM) were calculated by using TARQUIN and used to evaluate the level of noise. In addition, Cramér–Rao lower bound (CRLB) and CRLB percentage values were calculated to assess the reliability of metabolites. Quality control was then applied on prenoise suppression (preNS) ^1^H‐MRS in the following manner.

Exclusion criteria of ^1^H‐MRS cases are excluded if any of the following criteria is met.

1.: Histological diagnosis was missing.
2.: Water suppressed signals, water reference signals or structural MR images indicating the ^1^H‐MRS voxel location were missing.
3.: The tumour did not occupy all the ^1^H‐MRS voxel as determined by visual inspection of the voxel location images produced on the scanner aided by reference to the available image set.
4.: The spectrum showed FWHM larger than 0.15 ppm or 9.58 Hz for 1.5‐T ^1^H‐MRS.
5.: The spectrum showed fSNR smaller than 4.

For the cases that met these metric‐based quality measures, the TARQUIN‐processed ^1^H‐MRS spectrum of all cases was assessed visually by experienced spectroscopists (T.Z., J.T.G. and A.C.P.) for general quality features, namely phasing, fitting, baseline variation and the presence of artefacts [38].

### 2.5 Feature Extraction and Selection

Brain tumours were classified by combining ^1^H‐MRS and dMRI and compared against the results that were based on only one modality. Specifically, for ^1^H‐MRS, preNS and postnoise suppression (postNS) were additionally evaluated (Figure 1). When ^1^H‐MRS was involved, the metabolite profile of each individual patient was normalised based on the sum of all metabolites, lipid and macromolecule concentrations for tumour classification. When dMRI and ^1^H‐MRS were combined, ADC histogram features and metabolite concentrations were put together for ranking, screening and selection. The initial feature set included totally 27 ADC histogram features and 38 metabolites (Table S1), and all the features were ranked based on the multiclass area under the curve (mAUC) [17, 39]. Features were screened by two steps in order as (1) metabolites were removed if their CRLB percentage values were higher than 50% across all the cases and (2) metabolites or ADC histogram features were removed if they correlated to any of the remaining features (r>0.8, p<0.05) and had lower mAUC. The finally used features were obtained from the postscreening feature set by selecting those with the highest mAUC with a limit of no more than the sample size of the minority class minus one.

### 2.6 Tumour Classification

Tumour classification was performed by using conventional linear and non‐linear machine learning (ML) classifiers, namely, linear discriminant analysis (LDA), *k*‐nearest neighbours (kNN), naïve Bayes (NB), random forest (RF), multinomial log‐linear model fitting via a neural network (SLNN) and support vector machine (SVM) with a linear kernel. The probability that was derived through re‐substitution was used to show the certainty of tumour classification. LOOCV and *k*‐fold cross validation were used to determine tumour classification accuracies, where *k* was determined based on the size of the minority class. Both cross validation methods were performed to achieve some level of comparability, instead of selecting a more appropriate method for either cohort.

### 2.7 Statistical Analysis

The Mann–Whitney *U* test was performed to compare the demographic variables of patients between the three tumour types. The Pearson's *r* was used to evaluate the correlation between radiomic features. The Wilcoxon signed‐rank test was used to compare the brain tumour classification accuracies between modalities. Statistical significance values of the difference between classification accuracies were adjusted by using Bonferroni correction. All statistical analysis was performed by using R (Version 3.6.2, the R Foundation, Vienna, Austria).

**TABLE 4:** Demographic variables of the patients.

Tumour type	Sample size (M:F)	Age (mean ± SD, in years)
Ependymoma	6 (1:5)	6.4±6.9
Grade II	3 (1:2)	11.1±7.3
Tanycytic	1 (0:1)	15.3
Grade III	3 (0:3)	1.7±0.5
Anaplastic	2 (0:2)	1.9±0.5
Medulloblastoma	12 (7:5)	5.3±3.5
Desmoplastic nodular	2 (0:2)	8.0±9.3
Pilocytic astrocytoma	14 (5:9)	9.1±3.6

Abbreviations: F, female; M, male; SD, standard deviation.

## 3 Results

### 3.1 Demographics

Totally 32 patients (Table 4) who were diagnosed as ependymoma (N=6), medulloblastoma (N=12) or pilocytic astrocytoma (N=14) were involved in this study after screening. Demographic variables of patients did not show significant intertumour‐type difference (Table 4), except for the significantly elevated patient age of the pilocytic astrocytomas in comparison to the medulloblastomas (p<0.05, Mann–Whitney *U*).

**TABLE 5:** Selected apparent diffusion coefficient histogram features and metabolite concentrations used for classifying ependymomas, medulloblastomas and pilocytic astrocytomas.

Tumour type	EP	MB	PA	*p*	*p*	mAUC
	Mean ± SD	Mean ± SD	Mean ± SD	Raw	Normalised	Normalised
dMRI						
ADC 5th perc.	0.87±0.18	0.52±0.09	1.36±0.21	< 0.0001	/	0.970
ADC maximum	2.59±0.35	2.57±0.60	3.06±0.48	0.0237	/	0.721
ADC variance	60.97±44.16	86.22±46.95	69.09±38.39	0.4412	/	0.585
ADC entropy	0.10±0.04	0.09±0.04	0.09±0.04	0.7383	/	0.541
ADC kurtosis	9.27±7.00	8.31±4.31	9.00±7.14	0.9884	/	0.501
PreNS ^1^H‐MRS						
*myo*‐Inositol	9.98±5.58	1.57±1.77	1.66±1.29	0.0015	0.0005	0.888
Total LM at 0.9 ppm	3.30±1.79	7.20±4.11	3.94±1.69	0.0046	0.0011	0.862
Glx	7.01±1.91	5.19±1.95	4.96±1.86	0.0852	0.0008	0.840
Lactate	1.18±0.92	2.22±1.34	1.84±1.11	0.1546	0.0011	0.837
Total creatine	3.94±1.80	3.36±1.59	0.41±0.43	< 0.0001	0.0005	0.837
PostNS ^1^H‐MRS						
*myo*‐Inositol	13.45±6.85	1.02±1.74	2.99±2.25	< 0.0001	< 0.0001	0.952
Glx	6.64±1.67	5.97±2.25	6.34±3.84	0.6306	0.0003	0.853
Total creatine	5.28±2.47	3.86±2.23	0.90±1.28	0.0002	0.0021	0.826
Glycine	0.03±0.08	4.40±3.68	0.01±0.02	< 0.0001	< 0.0001	0.815
Total LM at 0.9 ppm	3.79±1.95	8.75±4.27	4.26±1.52	0.0014	0.0067	0.795

*Note:* Table showing the mean and standard deviation of the top five apparent diffusion coefficient (ADC) histogram features and metabolite concentrations that were extracted from either diffusion magnetic resonance imaging (dMRI) or proton magnetic resonance spectroscopy (^1^H‐MRS), where ^1^H‐MRS was comparatively shown as prenoise suppression or postnoise suppression. All ADC histogram features are in 10^‐3^mm^2^/s, and all metabolite concentrations and their combinations are in mM. The metabolite concentrations were calculated by using water signal as the reference, TARQUIN software and a ^1^H brain full basis. The *p* values were calculated by performing Mann–Whitney *U* test for the radiomic biomarkers between the three tumour groups. Specifically for spectroscopy, apart from the *p* values of the raw metabolite or metabolite combination concentrations, the *p* values of normalised metabolite or metabolite combination concentrations are also listed as *p* normalised.

Abbreviations: ^1^H‐MRS, proton magnetic resonance spectroscopy; ADC, apparent diffusion coefficient; dMRI, diffusion‐weighted magnetic resonance imaging; EP, ependymoma; Glx, glutamate and glutamine; LM, lipids and macromolecules; mAUC, multiclass area under the curve; MB, medulloblastoma; PA, pilocytic astrocytoma.

### 3.2 Spectroscopy Quality Assessment

The acquired ^1^H‐MRS had an overall fSNR as 16.5±8.9 and FWHM as 4.6±1.1 for all patients. The fSNR was significantly improved to be 22.3±11.4 after performing NS (p<0.05, Wilcoxon signed‐rank test), while FWHM was not significantly changed, being 4.7±1.2 (p>0.05, Wilcoxon signed‐rank test). The data showed no significant difference of spectral quality between the three tumour types (p>0.05, Kruskal–Wallis test, Table S8). Spectral quality was improved according to fSNR for every case (Table S8). Line width remains stable after NS.

### 3.3 Extracted Radiomic Features

**FIGURE 2:**
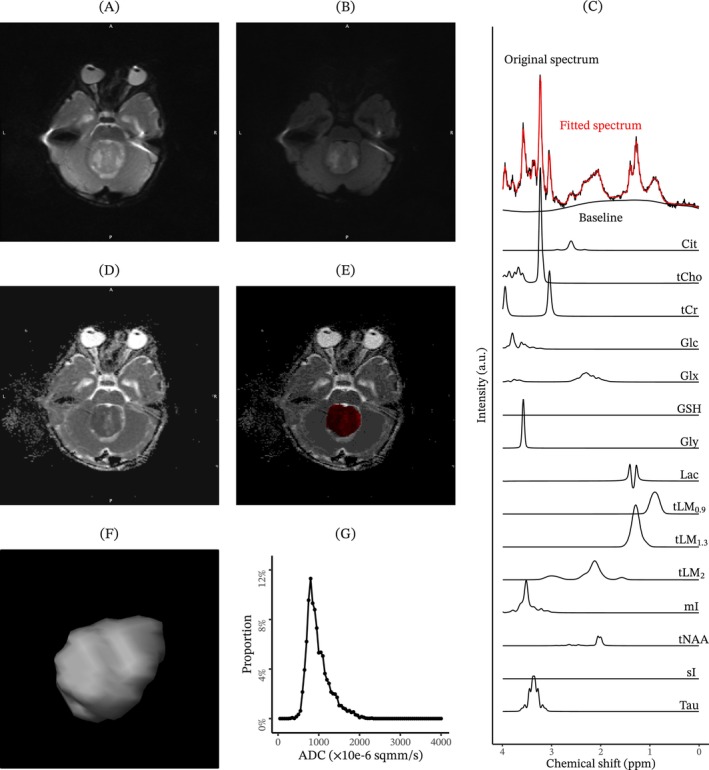
The acquired diffusion‐weighted magnetic resonance images and proton magnetic resonance spectroscopy for a randomly picked medulloblastoma case. *Note:* Images showing the acquired diffusion‐weighted magnetic resonance imaging (dMRI) for a randomly selected slice (A,B,D,E) and proton magnetic resonance spectroscopy of the localised single voxel (C). The dMRI example includes (A) the b0 image, (B) the b1000 image, the apparent diffusion coefficient image without (C) or with (D) a drawn region of interest for the tumour that is defined by experienced radiologists. In addition, (F) the reconstructed volume of interest for the tumour and the (G) apparent diffusion coefficient histogram are given in the end. proton magnetic resonance spectroscopy. Abbreviations: GSH, glutathione; Lac, lactate; mI, *myo*‐inositol; sI, *scyllo*‐inositol; Tau, taurine; tCho, total choline; tCr, total creatine; tLM_0.9_, total lipids and macromolecules at 0.9 ppm; tLM_1.3_, total lipids and macromolecules at 1.3 ppm; tLM_2_, total lipids and macromolecules at 2.0 ppm; tNAA, total *N*‐acetyl‐aspartate.

**FIGURE 3:**
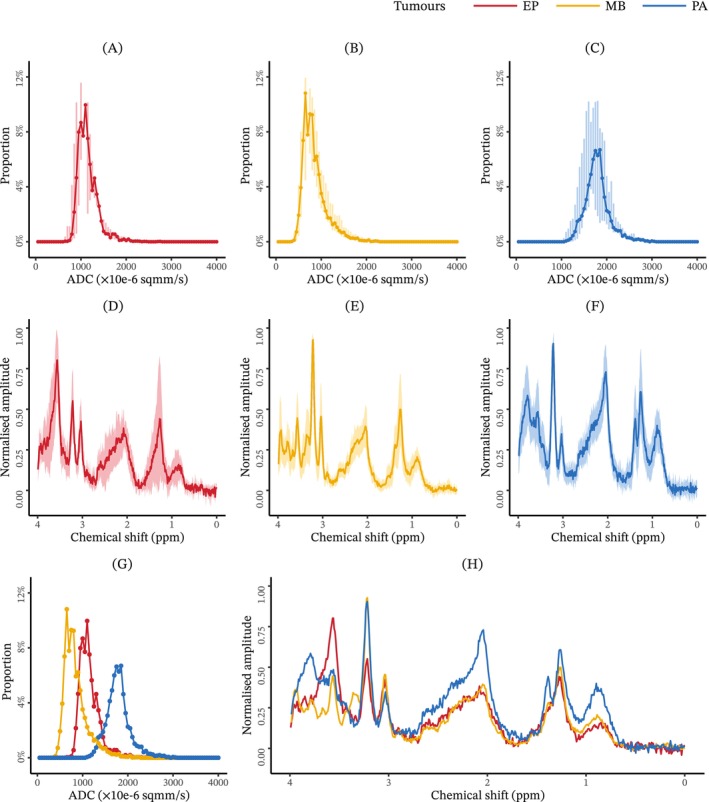
Line plots comparatively showing the median of apparent diffusion coefficient histograms or magnetic resonance spectra. *Note:* Images showing the median of apparent diffusion coefficient histograms (A–C,G) and postnoise suppression magnetic resonance spectra (D–F,H) for all the ependymomas (A,D), medulloblastomas (B,E), pilocytic astrocytomas (C,F) or all the patients in one plot but grouped by tumour types (G,H). In Subplots A–F, the black dots and lines denote the median of all cases in the corresponding tumour group, and the grey bars and area denote the standard deviation. In Subplots G and H, only the median values are provided. The median magnetic resonance spectra were shown by removing the estimated baseline and preserving the remaining spectral components including lipids and macromolecules.

Obtained dMRI images included b0 images, b1000 images and ADC images (Figure 2). Median VOI histogram showed visible difference between tumour types. ADC features showed generally higher water diffusion among pilocytic astrocytomas and lower water diffusion among medulloblastomas, while the ependymomas showed an intermediate degree of water diffusion. Median ^1^H‐MRS spectra showed tumour‐type‐wise differences (Figure 3).

Top ranked radiomic features from dMRI and ^1^H‐MRS after feature screening were obtained (Table 5). Among the features provided by dMRI (Table S2), ADC fifth percentile, ADC maximum (mAUC = 0.72), ADC variance (mAUC = 0.59), ADC entropy (mAUC = 0.54) and ADC kurtosis (mAUC = 0.50) had the highest diagnostic ability individually. Among the features provided by preNS ^1^H‐MRS (Table S2), *myo*‐inositol, tLM_0.9_, combined glutamate and glutamine, lactate and total creatine showed the highest diagnostic ability, while *myo*‐inositol, combined glutamate and glutamine, total creatine, glycine and tLM_0.9_ were provided by postNS ^1^H‐MRS. By combining the two modalities, the top five features became ADC minimum, *myo*‐inositol, tLM_0.9_, combined glutamate and glutamine and lactate for dMRI and preNS ^1^H‐MRS. Comparatively, ADC minimum, *myo*‐inositol, combined glutamate and glutamine, total creatine and glycine were given by dMRI and postNS ^1^H‐MRS.

### 3.4 Tumour‐Specific Classification

With the selected radiomic biomarkers, tumour‐specific classification accuracies varied between tumour types. The dMRI's classification accuracy (Table 6) is relatively lower for ependymomas (0%–67%) in comparison to ^1^H‐MRS (50%–83%). Combining dMRI and preNS ^1^H‐MRS does not seem to improve the classification accuracy for ependymomas very much (50%–83%), but combining dMRI and postNS ^1^H‐MRS can achieve higher accuracy (67%–100%). Medulloblastoma classification (Table 6) was relatively stable across different modalities. Comparatively, single‐modal methods achieved relatively more limited lower bound of accuracy (75%–100%) in comparison to multimodal methods (92%–100%). The dMRI achieved similar classification accuracies for pilocytic astrocytomas (79%–86%). The accuracy can be improved if using ^1^H‐MRS (79%–93%) and further when adding NS to ^1^H‐MRS (93%–100%). Multimodal methods achieved similar accuracy (93%–100%) to postNS ^1^H‐MRS.

### 3.5 Diagnostic Performance

Optimal tumour classification was achieved by combining dMRI and postNS ^1^H‐MRS, which outperformed over only one modality or combined dMRI and preNS ^1^H‐MRS. The used features were ADC fifth percentile, combined glutamate and glutamine, total creatine, glycine and tLM_0.9_, and the cross‐validated diagnostic accuracy was improved to 100% by using a NB classifier.

**FIGURE 4:**
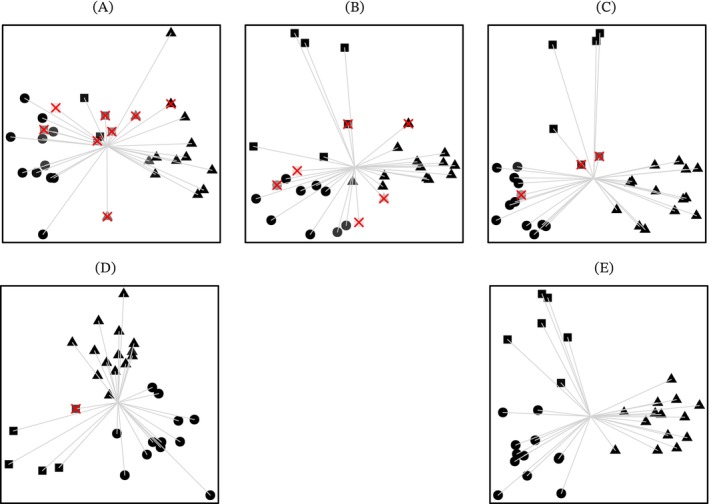
Scatter plots comparatively showing classification performance of different imaging methods. *Note:* Images showing the scatter plots where the three tumour types, ependymomas (in cubic), medulloblastomas (in triangle) and pilocytic astrocytomas (in circle), were classified. Decision tree results were generated as scatter plots with the two discriminant functions as *x* and *y* axes, where the discriminant functions are calculated by using the top five radiomic biomarkers from diffusion‐weighted magnetic resonance imaging and proton magnetic resonance spectroscopy. Radiomic biomarkers were supplied from (A) only dMRI, (B) only preNS ^1^H‐MRS, (C) only postNS ^1^H‐MRS, (D) both dMRI and preNS ^1^H‐MRS or (E) both dMRI and postNS ^1^H‐MRS.

Tumour classification performance showed a visible difference between imaging modalities (Figure 4), where classification hyperplanes showed clearer margins when combining the two imaging modalities. LOOCV suggested the balanced accuracy through dMRI to be up to 68%, which was achieved through NB. ^1^H‐MRS showed significantly higher classification accuracies (p<0.05, McNemar's with Bonferroni correction) in comparison to dMRI, and NS additionally improved its performance. LOOCV and six‐fold CV suggests that the balanced accuracy through postNS ^1^H‐MRS was significantly improved from both dMRI based and preNS ^1^H‐MRS based (p<0.05, McNemar's with Bonferroni correction). Taking SLNN as an example, the balanced classification accuracy determined through LOOCV was improved to 92% through postNS ^1^H‐MRS from 65% through dMRI or 78% through preNS ^1^H‐MRS. Multimodal imaging showed significantly higher accuracy than single‐modal imaging (P<0.05, McNemar's with Bonferroni correction). Among all the single and multimodal imaging methods, the combination of postNS ^1^H‐MRS and dMRI provides the optimal performance. The cross‐validated balanced accuracy was 100% through NB (Figure S4) by combining dMRI and postNS ^1^H‐MRS, in comparison to 68% through only dMRI, 81% through only preNS ^1^H‐MRS, 86% through combining dMRI and preNS ^1^H‐MRS and 94% through postNS ^1^H‐MRS. Six‐fold cross validation showed similar pattern for the five classifiers (Figures S2–S7).

**TABLE 6:** Cross‐validated brain tumour classification accuracies.

Tumour type	dMRI	PreNS ^1^H‐MRS	dMRI and preNS ^1^H‐MRS	PostNS ^1^H‐MRS	dMRI and postNS ^1^H‐MRS
Ependymomas	0%–67%	50%–83%	50%–83%	50%–83%	67%–100%
Medulloblastomas	75%–100%	75%–100%	75%–100%	92%–100%	92%–100%
Pilocytic astrocytomas	79%–86%	79%–93%	93%–100%	93%–100%	93%–100%
Overall	65%–87%	81%–94%	90%–94%	84%–97%	88%–100%
Balanced	56%–84%	76%–92%	83%–87%	81%–94%	84%–100%

*Note:* Table showing the leave‐one‐out cross‐validated brain tumour classification accuracies for specific tumour or all types. Accuracies were presented comparatively between MR modalities or modality combinations. The results were given as a range that summarises the six used machine learning classifiers (*k*‐nearest neighbours, linear discriminant analysis, naïve Bayes, neural network, random forest and support vector machine).

## 4 Discussion

This study presents a multimodal MRI/S‐based workflow that provides high‐accuracy paediatric brain tumour classification. Previous studies have identified radiomic biomarkers [20, 28] and designed novel computational models [15, 17] for either dMRI or ^1^H‐MRS, yet their results showed there was still space for further improvement in the diagnostic accuracy of ependymomas, medulloblastomas and pilocytic astrocytomas. In reality, clinical decisions are often made by a multidisciplinary team [40] after assessing MRI/S qualitatively, as preoperative scanning normally includes these sequences when available [41, 42]. All available imaging is discussed because the various sequences and modalities provide different insights to tumours through different imaging principles. Intuitively, multiple imaging modalities should be able to provide a more comprehensive demonstration of tumours, such as both cellular and metabolic characteristics. Histological classification may be linked to both cellularity and metabolism [43], as such a combination of dMRI and ^1^H‐MRS could be more representative to the molecular/pathological classification system than either on its own. This study confirms the feasibility and improved performance of these two modalities for brain tumour diagnosis, as well as emphasising the importance of the manner in which the ^1^H‐MRS processing and feature selection is undertaken.

### 4.1 ADCs

ADCs describe microscopic water diffusion in the presence of factors that restrict diffusion within tissues [44]. The use of ADCs or ADC histogram features in brain tumour classification has been reported in the last years, and they are considered as important quantitative imaging biomarkers for brain tumour diagnosis [21, 22, 25, 28]. Minimum ADCs were reported to be significantly correlated with tumour cellularity [45], but they do not seem to be better than mean ADCs [46], which means they can be used as the main dMRI biomarker. ADC percentile features were found to be significantly intercorrelated in this study (p<0.05) (Table S7), which may be because ADC percentile features describe histograms under similar mathematical principles with minor parameter differences [47]. Highly correlated features for classification lead to risks and are discouraged in data mining [48, 49]; thus, this study performed correlation analysis for ADC histogram features. In comparison to ADC fifth percentile (mAUC = 0.97), the other selected ADC histogram features had much lower diagnostic abilities. Visibly, the difference of median histograms between the three tumour types seems to be dominated by the shift of ADCs, where ependymomas are located between medulloblastomas and pilocytic astrocytomas (Figure 3). Considering the theory of histogram analysis [50], the diagnostic ability of ADCs for the three paediatric brain tumours may be mainly provided by only the one‐percentile feature that demonstrates the ADC shift of histograms.

This study excluded cysts when drawing tumour regions on dMRI. Although tumour‐related cyst lesions were reported to be distinguishable with dMRI [51], the usefulness of ADCs observed from cysts for paediatric posterior fossa tumour classification has not been clear. In line with previously reported pipelines [34], this study used cyst‐excluded tumour regions for the following analysis.

### 4.2 Metabolites

Metabolite profiles that are observed through non‐invasive ^1^H‐MRS can help paediatric brain tumour classification [5]. The ground truth of metabolite profiles may show intermetabolite correlations, because some metabolites are involved in the same metabolic pathways [52]. Yet, the observations of metabolite concentrations are affected by physical and mathematical concerns, such as scanning protocols [53] and quantification approaches [54], which makes the intercorrelation of metabolites more complicated. This study found little correlation between metabolites and between metabolites and ADC histogram features, which may indicate the two modalities share their individual and unique roles for brain tumour characterisation.

*myo*‐Inositol was found to have a high level in ependymomas according to previous ex vivo [55, 56] and in vivo [57, 58] studies, a finding which was observed in this study. Notably, glycine and *myo*‐inositol are relatively hard to distinguish in 1.5‐T in vivo ^1^H‐MRS because they share close resonances [59]. Therefore, the observed peak of *myo*‐inositol could be contributed by both *myo*‐inositol and glycine. According to a recent multifield‐strength study [17], glycine may be more distinguishable and significant over *myo*‐inositol for classifying the three tumour types in 3‐T in vivo ^1^H‐MRS. As in the 1.5‐T postNS ^1^H‐MRS, glycine seems to be elevated in medulloblastomas in comparison to the other tumour types, and at the same time, *myo*‐inositol remains significantly raised in ependymomas. This finding is supported by ex vivo magic angle spinning magnetic resonance spectroscopy [60, 61].

Lipids and macromolecules that are observed through short‐*T*_E_^1^H‐MRS are considered as biomarkers that may indicate tumour high grading [62] and poor survival [63]. This study found tLM_0.9_ to be a significant biomarker and particularly higher in medulloblastomas than the other two tumour types. Because medulloblastomas are classified as grade IV tumours [64] and are normally more aggressive than the other two types, this result may reflect the findings in the literature.

Combined glutamate and glutamine is often used in 1.5‐T in vivo ^1^H‐MRS because either of them is difficult to identify individually. Ependymomas were reported [20, 34] to have significantly higher level of combined glutamate and glutamine than other common paediatric posterior fossa tumours, which was observed in this study. It is noteworthy that combined glutamate and glutamine was lower, based on preNS ^1^H‐MRS, in pilocytic astrocytomas than in medulloblastomas, but according to postNS ^1^H‐MRS, this pattern was reversed. This observation may show the complexity and importance of combined glutamate and glutamine and its vulnerability to spectral noise when being used for diagnosing brain tumours.

Creatine is involved in cell cycle regulation and cellular energy metabolism [65], enabling it a potential biomarker for tumour characterisation. A finding that creatine was lower in pilocytic astrocytomas than ependymomas [57] may reflect this assumption. The use of creatine for characterising medulloblastomas remains unclear. This study suggests total creatine to be higher in ependymomas and lower in pilocytic astrocytomas, and the pattern is made clearer in postNS ^1^H‐MRS.

Observing lactate accurately through 1.5‐T short‐*T*_E_ PRESS‐localised spectroscopy is challenging, because the CH_3_ proton doublet resonance is weak and often mixed with lipids and macromolecules [66]. These issues make lactate a questionable metabolite for classifying tumours with 1.5‐T ^1^H‐MRS. PostNS ^1^H‐MRS seems to show lower diagnostic ability of lactate than preNS ^1^H‐MRS (Table 5). However, it is unclear whether this means NS provides more accurate diagnostic ability for lactate, because noise and spectral overlapping are theoretically unrelated.

### 4.3 Tumours

Different posterior fossa tumour types require different management. For instance, complete surgical resection is particularly important for the management of ependymoma. Consequently, accurate presurgical diagnosis is important for clinicians. Identifying ependymomas from other paediatric posterior fossa tumours by using in vivo metabolite profiles and ML was reported to have an accuracy at around 81% [25, 67]. Although the ADCs of ependymomas, which are higher than those of medulloblastomas and lower than those of pilocytic astrocytomas in this study, correspond to previous findings [10], the classification accuracies of ependymomas through only dMRI remain lower than other studies [25]. Comparatively, ependymomas gained more diagnostic precision from ^1^H‐MRS, where ^1^H‐MRS NS contributes to higher precision. Because ^1^H‐MRS is not always included in clinical practice [68], this study may encourage further clinical use of ^1^H‐MRS or multimodal methods.

Different posterior fossa tumour types require different management. For instance, complete surgical resection is particularly important for the management of ependymoma. Consequently, accurate presurgical diagnosis is important for clinicians. Identifying ependymomas from other paediatric posterior fossa tumours by using in vivo metabolite profiles and ML was reported to have an accuracy at around 81% [25, 67]. Although the ADCs of ependymomas, which are higher than those of medulloblastomas and lower than those of pilocytic astrocytomas in this study, correspond to previous findings [10], the classification accuracies of ependymomas through only dMRI remain lower than other studies [25]. Comparatively, ependymomas gained more diagnostic precision from ^1^H‐MRS, where ^1^H‐MRS NS contributes to higher precision. Because ^1^H‐MRS is not always included in clinical practice [68], this study may encourage further clinical use of ^1^H‐MRS or multimodal methods.

Characterising medulloblastomas through dMRI [28] or ^1^H‐MRS [69] has also been reported, especially for their subtyping [70, 71]. In this study, the diagnostic accuracy of medulloblastomas was not significantly different between dMRI and ^1^H‐MRS, and the combination of ^1^H‐MRS and dMRI was much better. When using only dMRI for tumour classification, misdiagnosis for medulloblastomas appears predominantly due to an unclear cut‐off of ADCs between medulloblastomas and ependymomas. Combining dMRI with ^1^H‐MRS helps overcome this problem (Table 5).

Similar to ependymomas, pilocytic astrocytomas were more accurately distinguished from the other two types when combining ^1^H‐MRS and dMRI, in comparison to using only one imaging modality. The main challenge of classifying pilocytic astrocytomas from the other two tumour types when using dMRI is their relatively similar ADCs to ependymomas for some cases. As with discriminating medulloblastoma and ependymoma this challenge could be overcome by combining dMRI with ^1^H‐MRS as there is significant difference between the two tumour types according to *myo*‐inositol, for example. The study also suggests that postNS ^1^H‐MRS alone may have good diagnostic ability for pilocytic astrocytomas and that the combination of dMRI and ^1^H‐MRS is similar. This may be a result of the relatively low cellularity of pilocytic astrocytomas which leads to a lower SNR which can be improved by NS.

### 4.4 Computational Approaches

Computational approaches in cancer imaging becomes increasingly important when considering translating imaging biomarker discoveries to clinical practice through radiomics [12, 72, 73]. It has been emphasised that computing plays a key role in data harmonisation [74], data reproducibility [75], image analysis [76] and radiomic analysis [77], and some of which addressed concerns from more than one discipline. Yet, computational approaches related to ML in MRS is still at an early stage [73], and most studies focus on physical advances in MRS (Table S9). For instance, principal component analysis remains the predominant feature analysis tool for MRS analysis, although it lacks transparency and explainability [73] (Table S10). To address these concerns, this study put previously designed computational models together for interpreting data and classifying tumours. The feature selection models used in this study inherited from the previous ^1^H‐MRS study [17], which was designed to make the tumour classification process transparent and interpretable [78].

Notably, the contribution of ^1^H‐MRS NS model to diagnostic performance seems to be clearer than adding dMRI. NS has not been considered to have an important role in the theoretical analysis of ^1^H‐MRS, based on the previously reported methods that rely on a good‐quality training set [79]. Surprisingly, this study showed more robust improvement of diagnostic performance when implementing NS model to ^1^H‐MRS rather than combining dMRI with preNS ^1^H‐MRS. The advantage that the NS model brings appears to result from the higher diagnostic ability of specific metabolites, such as *myo*‐inositol and combined glutamate and glutamine. Given the complexity and importance of these metabolites in the era of precision medicine [7], metabolite‐targeted research approaches may need to prioritised when considering clinical translation of ^1^H‐MRS. These results may imply the importance of performing advanced computational analysis on the data, in parallel to enriching the data source and optimising acquisition methods.

The study used multiple ML classifiers with conventional settings [67] to evaluate the performance, as the objective is to assess if combing dMRI and ^1^H‐MRS can bring better tumour classification performance than using only dMRI or ^1^H‐MRS. The ML classifiers used have their own underlying principles and are optimised for specific purposes. For instance, LDA [80] focuses on global optimisation by considering all cases when predicting borderline cases, while SVM [81] focuses on regional optimisation by considering mainly the cases that are close to borderline cases. It remains unclear about whether a specific ML classifier always outperforms the others for brain tumour MRI/S [73]. Therefore, the study assessed each classifier individually and summarised the performance by considering all classifiers, through which the results can show the advantage of using multimodal imaging and spectroscopy along with additional processing for ^1^H‐MRS.

Additionally, the study reported computational work by following regulations [82]. The performance of tumour classification was reported by diagnostic accuracy that is clinical meaningful. The used features for tumour classification were interpreted and compared with the literature.

### 4.5 Limitations and Future Work

This study has several limitations, primarily related to the methods of ADC feature extraction, the quantification of MRS and the limited tumour types and sample size.

ADC images were reconstructed by calculating the slope based on only b0 and b1000 images. A more accurate approach for generating ADC images could involve performing linear regression on b0, b500 and b1000 images or multishell diffusion imaging, although the current standard clinical practice is to use b0 and b1000.

Metabolite concentrations were estimated by using water signals as a reference. Following $T_{1}$ weighting and Gadolinium administration, there may have been slight variations in water signal [83, 84]. Consequently, the accuracy of metabolite concentration estimation may be affected, and the classification of brain tumours may be influenced by not only metabolite profiles but also contrast enhancement simultaneously. Although this method has demonstrated superior soundness compared to using creatine levels or the summation of all metabolite concentrations, due to the inherent differences between these levels across tumour types, it is acknowledged that the classification performance obtained in this study may have also been contributed by the variation in water signal between tissue types.

Future work includes expanding to a multicentre study and involving a wider range of tumour types, such as atypical teratoid rhabdoid tumours, and diffuse intrinsic pontine gliomas, and other rare embryonal tumours. Additional clinical validation in independent cohorts and through prospective cohort studies will be important steps in further evaluation and translation of this and other tumour classification pipelines.

## 5 Conclusions

Combining dMRI and ^1^H‐MRS can non‐invasively diagnose paediatric brain tumours accurately. Appropriate computational models of spectroscopy data processing and feature selection are necessary in such approaches.

## Conflicts of Interest

The authors declare no conflicts of interest.

## Supporting information



Supplementary.pdf

## Data Availability

The data that support the findings of this study are available on request from the corresponding author. The data are not publicly available due to privacy or ethical restrictions.
