# Supplementary material for: Accurate Paediatric Brain Tumour Classification Through Improved Quantitative Analysis of 1H MR Imaging and Spectroscopy
Source: NMR Biomed. 2025 Jul 23;38(9):e70103. doi: 10.1002/nbm.70103 (PMC12287626; doi:10.1002/nbm.70103)
Supplement: Supplementary file 1 — Supplementary.pdf [file NBM-38-e70103-s001.pdf]

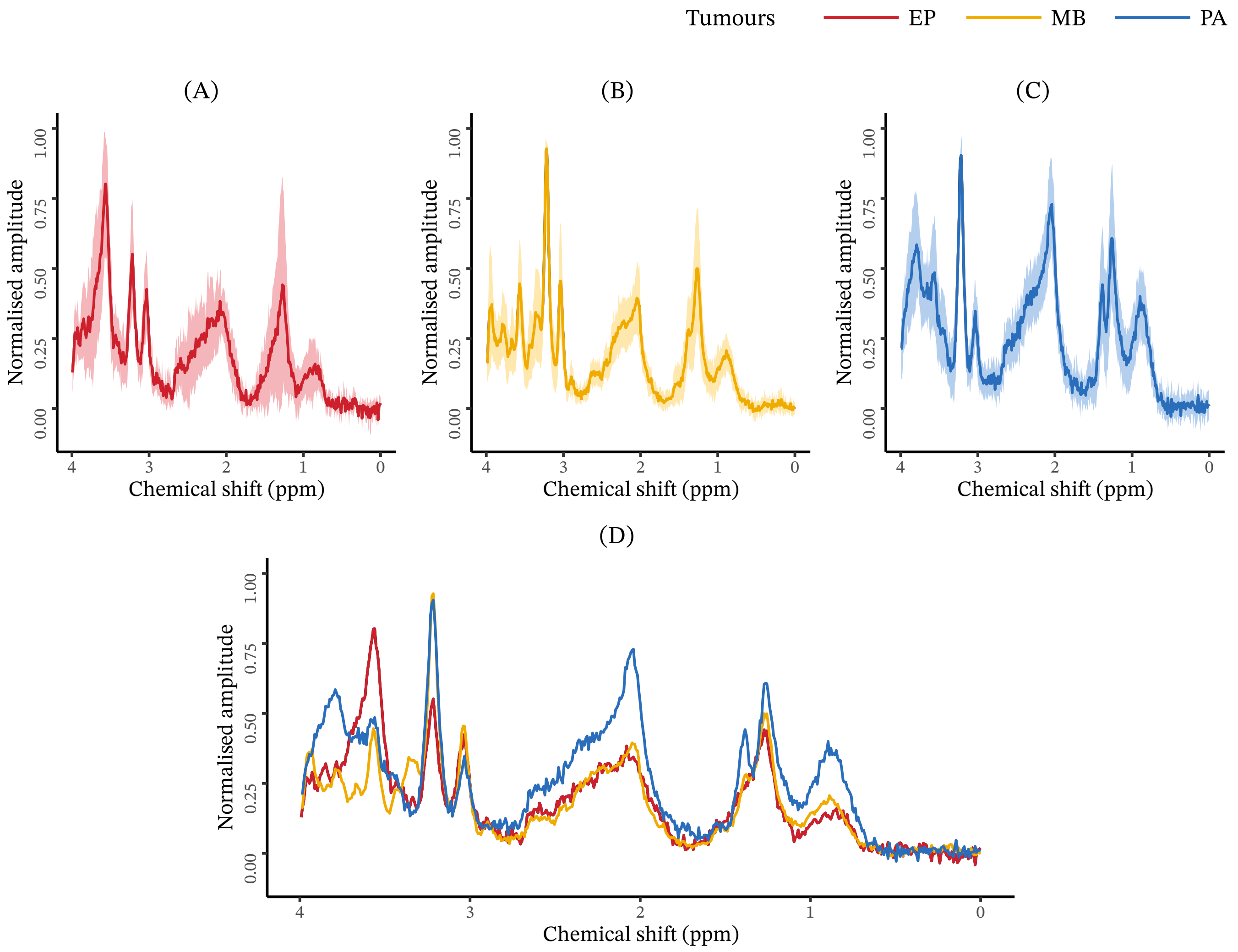

FIGURE 1 Line-plots comparatively showing the median of pre noise suppression magnetic resonance spectra.

Images showing the median of pre noise suppression magnetic resonance spectra (A–D) for all the ependymomas (A), medulloblastomas (B), pilocytic astrocytomas (C), or all the patients shown by the median (D). The median spectra were shown by removing the estimated baseline and preserving the remaining spectral components including lipids and macromolecules.

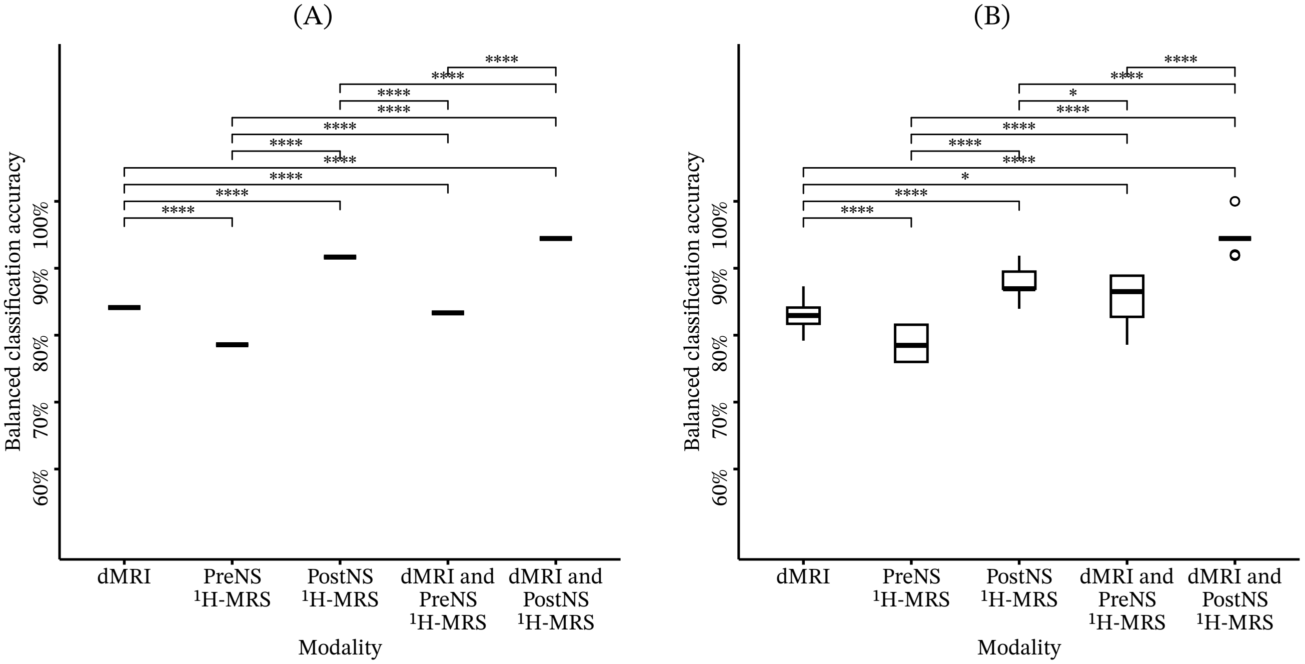

FIGURE 2 Classification accuracies according to *k*-nearest neighbours and leave-one-out (left) or six-fold (right) cross validation. *Abbreviations*: dMRI, diffusion-weighed magnetic resonance imaging; preNS, pre noise suppression; postNS, post noise suppression; ^{1}H-MRS, proton magnetic resonance spectroscopy; ns, not significant.

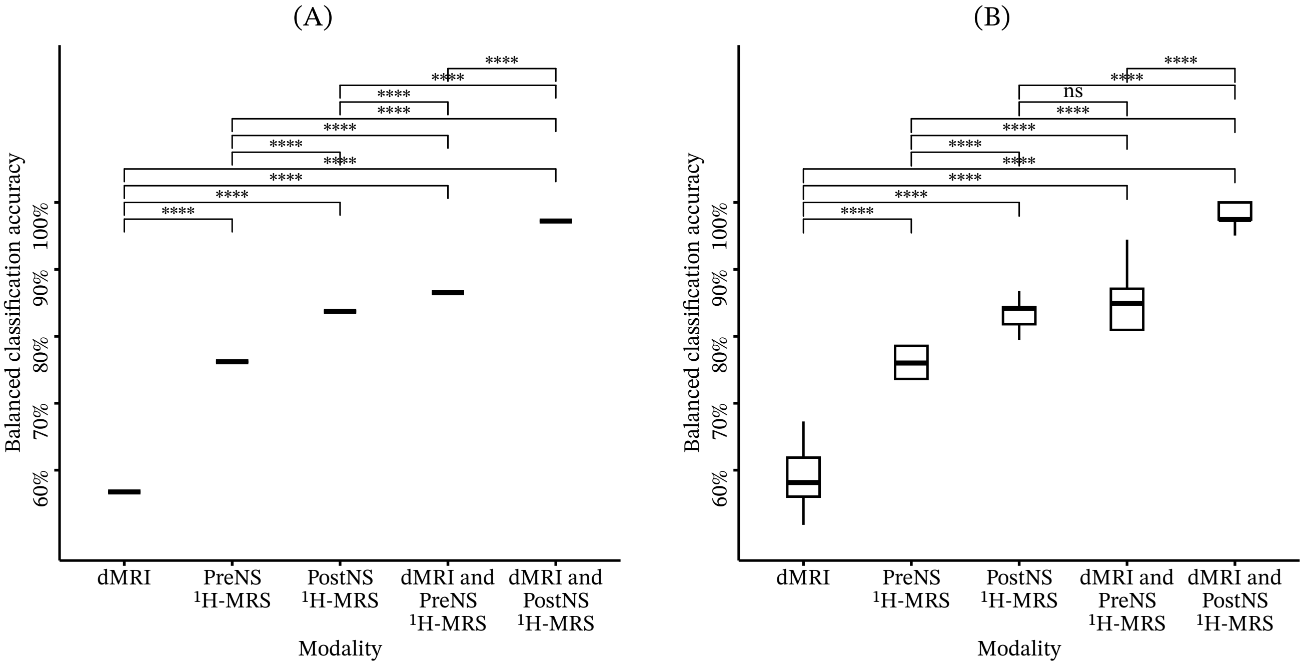

FIGURE 3 Classification accuracies according to linear discriminant analysis and leave-one-out (left) or six-fold (right) cross validation. *Abbreviations*: dMRI, diffusion-weighed magnetic resonance imaging; preNS, pre noise suppression; postNS, post noise suppression; ^{1}H-MRS, proton magnetic resonance spectroscopy; ns, not significant.

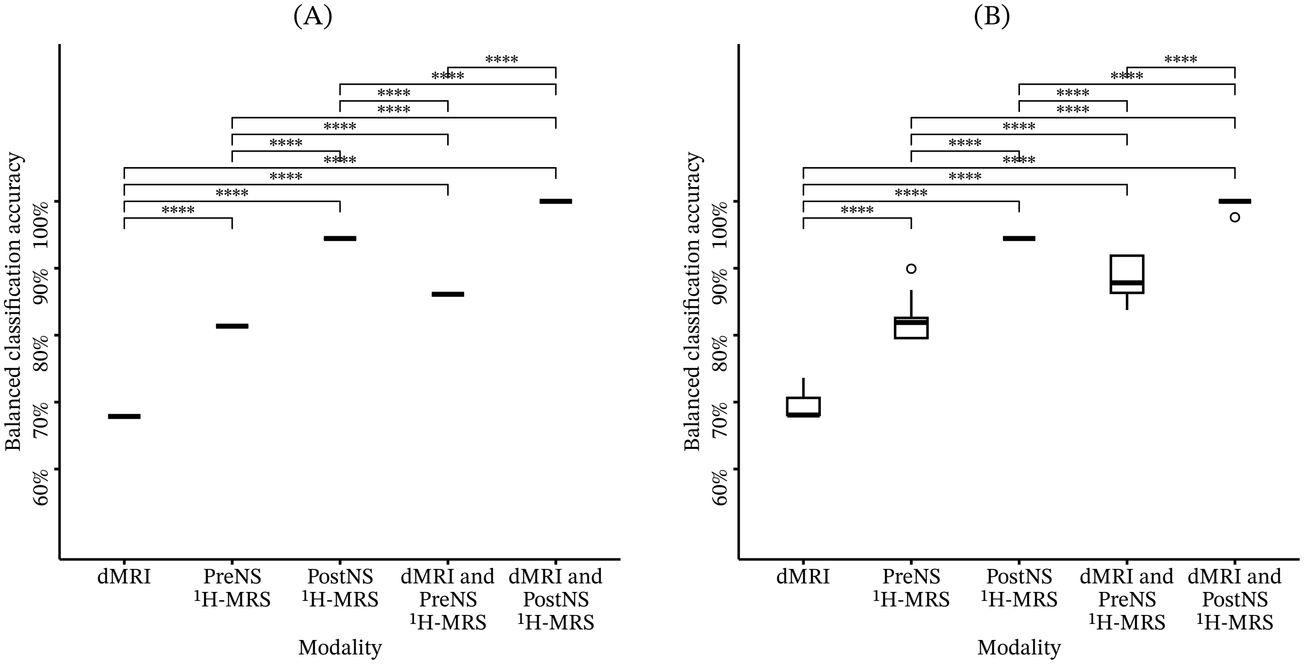

FIGURE 4 Classification accuracies according to naïve Bayes and leave-one-out (left) or six-fold (right) cross validation. *Abbreviations*: dMRI, diffusion-weighed magnetic resonance imaging; preNS, pre noise suppression; postNS, post noise suppression; ^{1}H-MRS, proton magnetic resonance spectroscopy; ns, not significant.

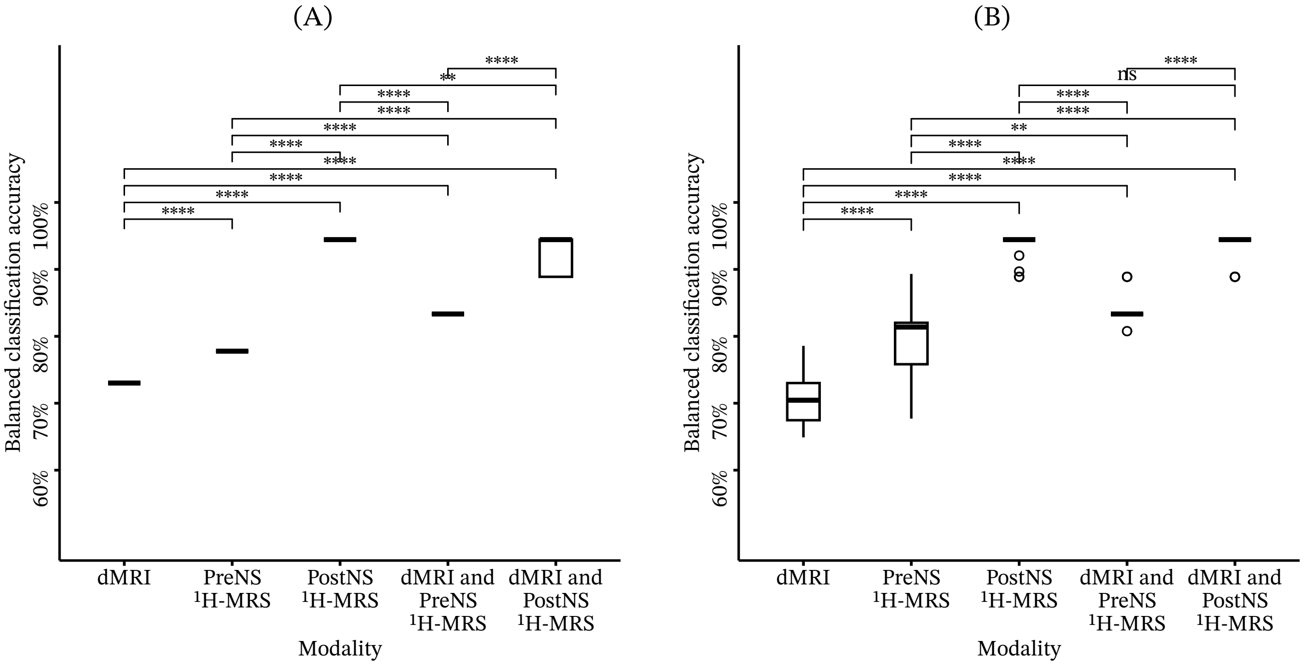

FIGURE 5 Classification accuracies according to random forest and leave-one-out (left) or six-fold (right) cross validation. *Abbreviations*: dMRI, diffusion-weighed magnetic resonance imaging; preNS, pre noise suppression; postNS, post noise suppression; ^{1}H-MRS, proton magnetic resonance spectroscopy; ns, not significant.

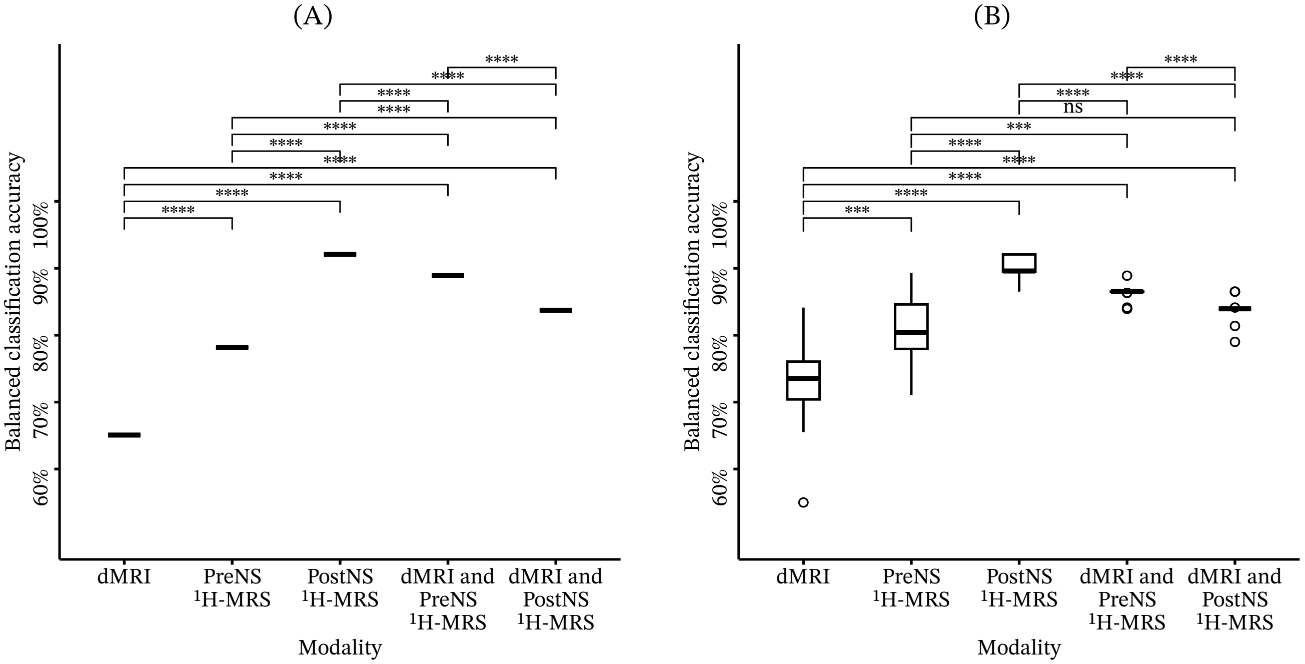

FIGURE 6 Classification accuracies according to neural network and leave-one-out (left) or six-fold (right) cross validation. *Abbreviations*: dMRI, diffusion-weighed magnetic resonance imaging; preNS, pre noise suppression; postNS, post noise suppression; ^{1}H-MRS, proton magnetic resonance spectroscopy; ns, not significant.

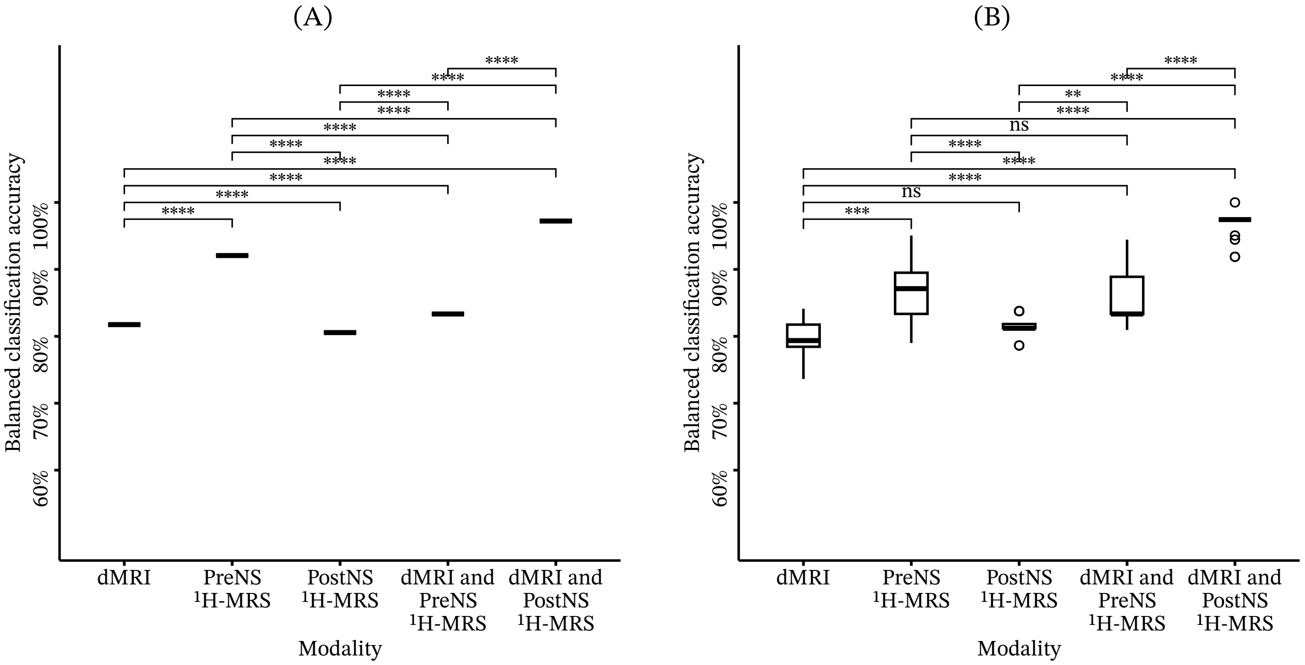

FIGURE 7 Classification accuracies according to support vector machine and leave-one-out (left) or six-fold (right) cross validation. *Abbreviations*: dMRI, diffusion-weighed magnetic resonance imaging; preNS, pre noise suppression; postNS, post noise suppression; ^{1}H-MRS, proton magnetic resonance spectroscopy; ns, not significant.

TABLE 1 Details of imaging and spectroscopic biomarker exclusion

Imaging/spectroscopic biomarker	Status	Reason of exclusion if applicable
Diffusion		
ADC 0th percentile (ADC minimum)	Included	/
ADC ith percentile ($i = 5, 10, 15, ..., 95$)	Excluded	Significantly correlated to ADC minimum
ADC 100th percentile (ADC maximum)	Included	/
ADC entropy	Included	/
ADC kurtosis	Included	/
ADC mean	Included	/
ADC mode	Included	/
ADC skewness	Included	/
ADC variance	Included	/
Spectroscopy		
Individual metabolites		
$-CrCH_2$	Excluded	CRLB percentage > 50% for all cases
Alanine (Ala)	Excluded	CRLB percentage > 50% for all cases
Aspartic acid (Asp)	Excluded	CRLB percentage > 50% for all cases
Citrate (Cit)	Included	/
Creatine, free (Cr)	Excluded	total creatine is used
γ-aminobutyric acid (GABA)	Excluded	CRLB percentage > 50% for all cases
Glycerophosphocholine (GPC)	Excluded	total choline is used
Glucose (Glc)	Excluded	CRLB percentage > 50% for all cases
Glutamate (Glu)	Excluded	combined glutamate and glutamine is used
Glutamine (Gln)	Excluded	combined glutamate and glutamine is used
Glutathione (GSH)	Included	/
Glycine (Gly)	Included	/
myo-Inositol	Included	/
scyllo-Inositol	Included	/
Lactate	Included	/
Lipids at 0.9 ppm ($Lip_{0.9}$)	Excluded	total lipids and macromolecules at 0.9 ppm is used
Lipids at 1.3 ppm, a ($Lip_{1.3,a}$)	Excluded	total lipids and macromolecules at 1.3 ppm is used
Lipids at 1.3 ppm, b ($Lip_{1.3,b}$)	Excluded	total lipids and macromolecules at 1.3 ppm is used
Lipids at 2.0 ppm ($Lip_{2.0}$)	Excluded	total lipids and macromolecules at 2.0 ppm is used
Macromolecules at 0.9 ppm ($MM_{0.9}$)	Excluded	total lipids and macromolecules at 0.9 ppm is used
Macromolecules at 1.2 ppm ($MM_{1.2}$)	Excluded	total lipids and macromolecules at 1.3 ppm is used
Macromolecules at 1.4 ppm ($MM_{1.4}$)	Excluded	total lipids and macromolecules at 1.3 ppm is used
Macromolecules at 1.7 ppm ($MM_{1.7}$)	Excluded	total lipids and macromolecules at 2.0 ppm is used
Macromolecules at 2.0 ppm ($MM_{2.0}$)	Excluded	total lipids and macromolecules at 2.0 ppm is used
Macromolecules at 3.8 ppm ($MM_{3.8}$)	Excluded	total lipids and macromolecules at 2.0 ppm is used
N-acetyl-aspartate (NAA)	Excluded	total *N*-acetyl-aspartate is used
N-acetyl-aspartyl-glutamate (NAAG)	Excluded	total *N*-acetyl-aspartate is used
Phosphocholine (PCho)	Excluded	total choline is included
Phosphocreatine (PCr)	Excluded	total creatine is included
Phosphatidylethanol (PEth)	Excluded	CRLB percentage > 50% for all cases
Taurine	Included	/
Metabolite combinations		
total Choline (tCho)	Included	/
total Creatine (tCr)	Included	/
combined Glutamate and glutamine (Glx)	Included	/
total Lipids and macromolecules at 0.9 ppm ($tLM_{0.9}$)	Included	/
total Lipids and macromolecules at 1.3 ppm ($tLM_{1.3}$)	Included	/
total Lipids and macromolecules at 2.0 ppm ($tLM_{2.0}$)	Included	/
total *N*-acetyl-aspartate (tNAA)	Included	/

TABLE 2 Diagnostic ability of eligible radiomic biomarkers for selection from dMRI and ^{1}H-MRS

#	dMRI and preNS ^{1}H-MRS		dMRI and postNS ^{1}H-MRS	
	**Imaging/metabolic biomarker**	**multi-class AUC**	**Imaging/metabolic biomarker**	**multi-class AUC**
1	ADC minimum	.976	ADC minimum	.976
2	*myo*-Inositol	.887	*myo*-Inositol	.947
3	total LM at 0.9 ppm	.847	Glx	.861
4	Glx	.843	total Creatine	.822
5	Lactate	.839	Glycine	.815
6	total Creatine	.836	Total LM at 0.9 ppm	.783
7	Glucose	.788	Lactate	.782
8	total LM at 1.3 ppm	.773	Citrate	.778
9	total *N*-Acetyl-Aspartate	.745	total Choline	.744
10	Glycine	.729	total LM at 1.3 ppm	.740
11	ADC maximum	.710	total NAA	.737
12	total Choline	.698	ADC maximum	.710
13	Citrate	.662	total LM at 2.0 ppm	.685
14	Glutathione	.641	Glucose	.684
15	Taurine	.632	Taurine	.620
16	total LM at 2.0 ppm	.587	Glutathione	.617
17	ADC variance	.579	ADC variance	.579
18	ADC kurtosis	.517	ADC kurtosis	.517

TABLE 3 Correlation between apparent diffusion coefficient histogram features.

	Mean	Var	Max	Min	Skew	Mode	Kurt	Ent	P_{10}	P_{20}	P_{30}	P_{40}	P_{50}	P_{60}	P_{70}	P_{80}
Var	.04															
Max	.53	.30														
Min	.86	−.25	.43													
Skew	−.24	−.26	.36	.04												
Mode	.97	−.10	.49	.85	−.24											
Kurt	−.09	−.41	.34	.14	.86	−.05										
Ent	.19	.00	.58	.21	.37	.19	.46									
P_{10}	.97	−.16	.51	.92	−.09	.97	.05	.23								
P_{20}	.98	−.12	.51	.90	−.15	.98	.01	.22	.99							
P_{30}	.99	−.08	.51	.88	−.20	.99	−.03	.20	.99	.99						
P_{40}	.99	−.04	.50	.87	−.23	.99	−.06	.18	.98	.99	.99					
P_{50}	.99	.00	.50	.86	−.27	.98	−.09	.17	.97	.99	.99	.99				
P_{60}	.99	.04	.49	.84	−.30	.97	−.13	.16	.96	.98	.99	.99	.99			
P_{70}	.99	.10	.49	.82	−.32	.96	−.16	.15	.94	.96	.98	.99	.99	.99		
P_{80}	.99	.18	.51	.80	−.33	.94	−.20	.15	.92	.94	.96	.97	.98	.99	.99	
P_{90}	.96	.30	.54	.75	−.34	.90	−.26	.15	.88	.90	.91	.93	.94	.96	.97	.99

Table showing the Pearson's r of the apparent diffusion coefficient histogram features based on the whole cohort.
Accessed apparent diffusion coefficient (ADC) features include ADC mean (Mean), ADC variance (Var), ADC maximum (Max), ADC minimum (Min), ADC skewness (Skew), ADC mode (Mode), ADC kurtosis (Kurt), ADC entropy (Ent), and i-th percentile of the ADC ($i = 10, 20, ..., 90$).

TABLE 4 The Pearson's cross correlation coefficients between metabolites in pre noise suppression magnetic resonance spectroscopy.

	Cit	Glc	Gly	GSH	mI	Lac	Tau	tNAA	tCho	tCr	Glx	$tLM_{0.9}$	$tLM_{1.3}$
Glc	0.16												
Gly	0.26	−0.26											
GSH	0.46*	−0.34	0.19										
mI	0.37*	0.66*	−0.03	−0.06									
Lac	0.02	−0.35	0.11	0.30	−0.41*								
Tau	0.25	−0.17	0.17	0.33	−0.01	−0.15							
tNAA	0.29	0.16	0.03	−0.03	0.39*	−0.28	−0.23						
tCho	0.23	−0.44*	0.50*	0.41*	−0.02	0.09	0.38*	−0.16					
tCr	0.56*	−0.20	0.45*	0.48*	0.22	−0.12	0.53*	0.19	0.63*				
Glx	0.52*	−0.06	−0.03	0.43*	0.22	0.22	−0.14	0.43*	−0.05	0.22			
$tLM_{0.9}$	0.04	−0.41*	0.23	0.41*	−0.29	0.78*	0.07	−0.22	0.20	0.00	0.22		
$tLM_{1.3}$	0.13	−0.36*	0.23	0.45*	−0.24	0.75*	−0.01	−0.19	0.17	0.05	0.31	0.95*	
$tLM_{2.0}$	0.51*	−0.22	0.42*	0.48*	0.29	0.27	0.38*	0.03	0.60*	0.61*	0.29	0.58*	0.59*

Table showing the Pearson's *r* of the metabolite concentrations based on the whole cohort.
Accessed metabolites include Citrate (Cit), total Choline (tCho), total Creatine (tCr), Glucose (Glc), Glycine (Gly), Glutathione (GSH), Lactate (Lac), total lipids and macromolecules at 0.9 ppm ($tLM_{0.9}$), total lipids and macromolecules at 1.3 ppm ($tLM_{1.3}$), total lipids and macromolecules at 2.0 ppm ($tLM_{2.0}$), total *N*-acetyl-aspartate (tNAA), *myo*-Inositol (mI), and Taurine (Tau).

TABLE 5 The Pearson's cross correlation between metabolites in post noise suppression magnetic resonance spectroscopy.

	Cit	Glc	Gly	GSH	mI	Lac	Tau	tNAA	tCho	tCr	Glx	$tLM_{0.9}$	$tLM_{1.3}$
Glc	−0.04												
Gly	0.16	0.01											
GSH	0.21 ↓	0.04	0.60*↑										
mI	0.42*	0.06 ↓	−0.07	−0.28									
Lac	−0.08	−0.08	−0.03	0.35	−0.44*								
Tau	0.11	−0.11	0.63*↑	0.36*↑	0.11	−0.08							
tNAA	0.32	0.31	0.15	0.08	−0.01 ↓	−0.05	0.02						
tCho	0.39*	−0.16 ↓	0.61*	0.55*	−0.04	0.20	0.53*	0.16					
tCr	0.61*	0.11	0.54*	0.36*	0.32	−0.11	0.64*	0.17	0.53*				
Glx	0.21	−0.09	0.01	0.14 ↓	0.20	−0.01	0.05	0.15 ↓	−0.07	0.33			
$tLM_{0.9}$	−0.01	−0.19 ↓	0.31	0.65*	−0.35	0.58*	0.19	−0.12	0.41*↑	0.07	0.11		
$tLM_{1.3}$	0.02	−0.23 ↓	0.19	0.58*	−0.28	0.51*	0.02	−0.20	0.20	0.05	0.21	0.90*	
$tLM_{2.0}$	0.42*	−0.10	0.56*	0.65*	0.21	0.13	0.63*	−0.06	0.72*	0.63*	0.26	0.59*	0.43*

Table showing the Pearson's *r* of the metabolite concentrations based on the whole cohort.
Accessed metabolites include Citrate (Cit), total Choline (tCho), total Creatine (tCr), Glucose (Glc), Glycine (Gly), Glutathione (GSH), Lactate (Lac), total lipids and macromolecules at 0.9 ppm ($tLM_{0.9}$), total lipids and macromolecules at 1.3 ppm ($tLM_{1.3}$), total lipids and macromolecules at 2.0 ppm (tLM-subscript2.0), total *N*-acetyl-aspartate (tNAA), *myo*-Inositol (mI), and Taurine (Tau). Correlation coefficients that were non-significant prior to noise suppression but became significant afterward were marked with ↑; those that lost significance following noise suppression were marked with ↓.

TABLE 6 Correlation between apparent diffusion coefficient histogram features and pre noise suppression metabolites or metabolite combinations.

	Mean	Variance	Max	Min	Skewness	Mode	Kurtosis	Entropy	Perc25	Perc50	Perc75
Cit	−0.30	−0.14	−0.46*	−0.27	−0.24	−0.30	−0.21	−0.13	−0.30	−0.29	−0.27
Glc	0.23	−0.36*	0.02	0.45*	0.22	0.23	0.26	0.04	0.27	0.24	0.19
GSH	−0.38*	0.07	−0.14	−0.38*	−0.10	−0.36*	−0.14	−0.01	−0.39*	−0.37*	−0.35
Gly	−0.16	0.35	−0.20	−0.31	−0.33	−0.21	−0.35	0.13	−0.23	−0.16	−0.09
mI	−0.15	−0.28	−0.17	0.00	0.17	−0.12	0.21	0.16	−0.11	−0.14	−0.17
Lac	−0.04	0.13	0.08	−0.09	−0.04	−0.04	−0.16	0.03	−0.06	−0.05	−0.02
Tau	−0.42*	0.21	−0.24	−0.52*	−0.01	−0.46*	−0.14	−0.08	−0.46*	−0.43*	−0.40*
tNAA	−0.06	−0.22	−0.21	0.00	0.11	−0.02	0.19	0.04	−0.03	−0.05	−0.07
tCho	−0.60*	0.05	−0.20	−0.58*	0.09	−0.60*	−0.04	0.10	−0.60*	−0.60*	−0.58*
tCr	−0.62*	0.00	−0.44*	−0.62*	0.00	−0.65*	−0.07	−0.19	−0.64*	−0.61*	−0.59*
Glx	−0.03	−0.22	−0.32	−0.02	−0.18	−0.01	−0.07	0.08	−0.01	−0.02	−0.03
tLM09	−0.29	0.15	−0.04	−0.28	0.02	−0.24	−0.10	0.10	−0.30	−0.30	−0.27
tLM13	−0.32	0.12	−0.07	−0.30	−0.01	−0.27	−0.07	0.09	−0.33	−0.32	−0.30
tLM20	−0.76*	−0.01	−0.41*	−0.69*	0.06	−0.72*	−0.06	0.04	−0.75*	−0.75*	−0.74*
Total	−0.50*	0.08	−0.25	−0.48*	−0.01	−0.47*	−0.10	0.11	−0.52*	−0.50*	−0.47*
TotalExcludeLM	−0.48*	−0.02	−0.42*	−0.47*	−0.05	−0.49*	−0.10	0.10	−0.49*	−0.48*	−0.45*

Pearson's correlation coefficient (r) between ADC histogram features and pre noise suppression metabolite concentrations across the whole cohort.
Metabolite abbreviations: Cit (Citrate), Glc (Glucose), GSH (Glutathione), Gly (Glycine), mI (*myo*-Inositol), Lac (Lactate), Tau (Taurine), tNAA (total *N*-acetyl-aspartate), tCho (total Choline), tCr (total Creatine), Glx (Glutamate + Glutamine), tLM09, tLM13, tLM20 (Lipids + Macromolecules at 0.9, 1.3, and 2.0 ppm).
ADC histogram features: mean, variance, maximum, minimum, skewness, mode, kurtosis, entropy, and 25th/50th/75th percentiles.
***** indicates statistical significance ($P < 0.05$).

TABLE 7 Correlation between apparent diffusion coefficient histogram features and post noise suppression metabolites or metabolite combinations.

	Mean	Variance	Max	Min	Skewness	Mode	Kurtosis	Entropy	Perc25	Perc50	Perc75
Cit	−0.30	−0.09	−0.34	−0.29	0.00	−0.30	0.01	0.03	−0.30	−0.29	−0.28
Glc	0.17	0.06	0.06	0.27	0.10	0.08	0.09	−0.24	0.14	0.17	0.17
Gly	−0.49*	0.23	−0.36*	−0.50*	−0.17	−0.51*	−0.23	−0.10	−0.54*	−0.48*	−0.43*
GSH	−0.39*	0.29	−0.11	−0.48*	−0.20	−0.44*	−0.23	0.04	−0.44*	−0.39*	−0.33
mI	0.07	−0.25	0.00	0.15	0.13	0.09	0.20	0.29	0.10	0.08	0.05
Lac	−0.18	0.13	0.03	−0.23	0.07	−0.18	−0.06	0.06	−0.19	−0.19	−0.17
Tau	−0.48*	0.19	−0.10	−0.56*	0.17	−0.50*	0.03	0.12	−0.50*	−0.49*	−0.46*
tNAA	0.05	−0.09	−0.09	0.02	−0.05	0.06	0.06	−0.11	0.06	0.06	0.05
tCho	−0.68*	−0.02	−0.27	−0.64*	0.13	−0.67*	0.01	0.05	−0.68*	−0.68*	−0.68*
tCr	−0.49*	0.03	−0.29	−0.50*	0.11	−0.53*	0.06	−0.09	−0.51*	−0.49*	−0.47*
Glx	0.09	−0.24	0.02	0.18	0.13	0.11	0.19	0.14	0.13	0.10	0.07
tLM09	−0.44*	0.11	−0.11	−0.39*	0.14	−0.41*	−0.03	0.08	−0.45*	−0.46*	−0.44*
tLM13	−0.39*	0.09	−0.12	−0.35	0.04	−0.34	−0.04	0.08	−0.40*	−0.39*	−0.37*
tLM20	−0.63*	0.10	−0.29	−0.57*	0.18	−0.65*	0.02	0.13	−0.64*	−0.64*	−0.61*
Total	−0.57*	0.06	−0.23	−0.51*	0.13	−0.55*	0.02	0.14	−0.58*	−0.57*	−0.55*
TotalExcludeLM	−0.44*	−0.05	−0.24	−0.41*	0.14	−0.46*	0.10	0.15	−0.45*	−0.44*	−0.43*

Pearson's correlation coefficient (r) between ADC histogram features and post noise suppression metabolite concentrations across the whole cohort.
Metabolite abbreviations: Cit (Citrate), Glc (Glucose), GSH (Glutathione), Gly (Glycine), mI (*myo*-Inositol), Lac (Lactate), Tau (Taurine), tNAA (total *N*-acetyl-aspartate), tCho (total Choline), tCr (total Creatine), Glx (Glutamate + Glutamine), tLM09, tLM13, tLM20 (Lipids + Macromolecules at 0.9, 1.3, and 2.0 ppm).
ADC histogram features: mean, variance, maximum, minimum, skewness, mode, kurtosis, entropy, and 25th/50th/75th percentiles.
***** indicates statistical significance ($P < 0.05$).

TABLE 8 Details of MR image and spectroscopy acquisition protocols

	Manufacturer	NCC	FS (Hz)	TE (ms)	TR (ms)	NCP	VS (mm^3)	fSNR preNS	fSNR postNS	FWHM preNS (Hz)	FWHM postNS (Hz)
Ependymoma											
#1	GE Signa	8	2000	30	1500	2048	20×20×20	6.9	9.1	4.0	3.7
#2	Siemens Avanto	12	2000	30	1500	2048	15×15×15	9.2	12.7	5.4	7.3
#3	GE Signa	8	2500	30	1500	2048	15×15×15	11.1	14.6	4.0	4.8
#4	Siemens Aera	20	1000	30	2200	1024	15×15×15	22.7	34.6	5.4	5.1
#5	Siemens Aera	20	1000	30	2200	1024	15×15×15	25.7	38.3	5.4	5.4
#6	Siemens Avanto	12	2000	30	1500	2048	20×20×20	32.2	39.4	5.1	5.1
Medulloblastoma											
#7	Siemens Avanto	12	2000	30	1500	2048	15×15×15	8.4	10.8	7.6	7.1
#8	GE Signa	8	2500	30	1500	2048	15×15×15	8.5	9.8	4.4	4.2
#9	Siemens Avanto	12	2000	30	1500	2048	15×15×15	13.4	19.6	3.1	3.3
#10	Siemens Avanto	12	2000	30	1500	2048	20×20×20	14.2	20.3	4.1	4.1
#11	Siemens Avanto	12	2000	30	1500	2048	20×20×20	21.2	28.7	3.6	3.6
#12	Siemens Avanto	12	2000	30	1500	2048	20×20×20	21.9	32.5	2.9	3.4
#13	Siemens Symphony	16	2000	30	1500	2048	20×20×20	25.7	34.9	3.9	3.9
#14	Siemens Symphony	16	2000	30	1500	2048	20×20×20	25.7	37.3	3.7	3.7
#15	Siemens Avanto	12	2000	30	1500	2048	15×15×15	27.1	39.7	3.7	3.9
#16	Siemens Avanto	12	2000	30	1500	2048	20×20×20	27.5	33.0	4.2	4.2
#17	Siemens Avanto	12	2000	30	1500	2048	20×20×20	32.2	36.5	3.1	3.1
#18	Siemens Avanto	12	2000	30	1500	2048	20×20×20	38.5	46.4	3.2	3.2
Pilocytic astrocytoma											
#19	Siemens Avanto	12	2000	30	1500	2048	15×15×15	5.8	8.7	6.3	5.9
#20	Siemens Avanto	12	2000	30	1500	2048	15×15×15	6.4	9.8	5.1	4.9
#21	Siemens Symphony	16	2000	30	1500	2048	15×15×15	7.6	12.3	6.1	7.3
#22	Siemens Avanto	12	2000	30	1500	2048	15×15×15	8.2	12.5	5.1	5.3
#23	Siemens Avanto	12	2000	30	1500	2048	20×20×20	8.5	10.6	5.1	7.8
#24	Siemens Avanto	12	2000	30	1500	2048	20×20×20	10.1	14.3	4.4	4.4
#25	Siemens Avanto	12	2000	30	1500	2048	20×20×20	10.6	14.2	5.9	5.6
#26	Siemens Symphony	16	2000	30	1500	2048	20×20×20	11.0	13.6	4.2	4.2
#27	Siemens Symphony	16	2000	30	1500	2048	20×20×20	11.1	15.8	5.4	5.1
#28	Siemens Avanto	12	2000	30	1500	2048	15×15×15	12.6	17.0	4.4	4.4
#29	Siemens Avanto	12	2000	30	1500	2048	15×15×15	13.5	18.3	5.1	5.1
#30	Siemens Symphony	16	2000	30	1500	2048	20×20×20	15.1	23.2	4.6	4.6
#31	Siemens Avanto	12	2000	30	1500	2048	20×20×20	16.3	21.0	3.7	3.9
#32	Siemens Symphony	16	2000	30	1500	2048	20×20×20	18.1	23.6	3.9	3.9

Abbreviations: NCC, number of head coil channels; FS, frequency of sampling; TE, time of echo; TR, time of repetition; NCP, number of complex points; VS, voxel size; fSNR, fitting-based signal-to-noise ratio; FWHM, full-width at half maximum; preNS, pre noise suppression; postNS, post noise suppression.

TABLE 9 Details of image and spectroscopy acquisition and analysis parameters.

Authors	Year	Scanning Protocol			
		B_0 (T)	T_E (ms)	T_R (ms)	Sequence
dMRI					
Yamasaki et al.[1]	2005	1.5	107	1600	EPI
Rumboldt et al.[2]	2006	1.5	*not reported*	*not reported*	*not reported*
Yamashita et al.[3]	2009	1.5	72	5000	EPI
Bull et al.[4]	2012	1.5	96–107	2700–3600	EPI
R. Gutierrez et al.[5]	2014	1.5	59–89	4883–5800	*not reported*
Novak et al.[6]	2021	1.5, 3.0	60–147	2435–8575	*not reported*
^{1}H-MRS					
Davies et al.[7]	2008	1.5	30	1500	PRESS
Raschke et al.[8]	2012	1.5	30	1500	PRESS
Vicente et al.[9]	2013	1.5	20–30	1500–2000	PRESS, PROBE, STEAM
		1.5	135–136	1500–2020	PRESS
Zarinabad et al.[10]	2017	1.5	30	1500	PRESS
Zarinabad et al.[11]	2018	3.0	30–46	2000	PRESS
Manias et al.[12]	2018	1.5	28–35	1500	PRESS
Davies et al.[7]	2022	1.5	30	1500	PRESS
Zhao et al.[13]	2022	1.5, 3.0	30	1500	PRESS
Zhao et al.[14]	2024	1.5, 3.0	30–46	1500–2000	PRESS
dMRI and ^{1}H-MRS					
Schneider et al.	2007	1.5	dMRI 107, ^{1}H-MRS 30	dMRI 3600, ^{1}H-MRS 1500	EPI, PRESS

Abbreviations: ^{1}H-MRS, proton magnetic resonance spectroscopy; B_0, field strength; EPI, echo-planar imaging; dMRI, diffusion-weighted magnetic resonance imaging; PRESS, point resolved spectroscopy; PROBE, standard GE sequence for single-voxel spectroscopy; STEAM, stimulated echo acquisition mode; T_E, echo time; T_R, repetition time.

TABLE 10 Details of image and spectroscopy acquisition and analysis parameters.

Authors	Year	Pre-Processing	Extracted Features	Feature Selection	Classifier
dMRI					
Yamasaki et al.[1]	2005	*not reported*	Raw ADCs	*not reported*	*not reported*
Rumboldt et al.[2]	2006	*not reported*	Raw ADCs	*not reported*	*not reported*
Yamashita et al.[3]	2009	*not reported*	ADC features	Manual	*not reported*
Bull et al.[4]	2012	*not reported*	ADC features	Manual	Linear regression
R. Gutierrez et al.[5]	2014	*not reported*	ADC features	Manual	SVM
Novak et al.[6]	2021	*not reported*	ADC features	PCA	NB,RF
^{1}H-MRS					
Davies et al.[7]	2008	*not reported*	Metabolite concentrations	PCA	LDA
Raschke et al.[8]	2012	*not reported*	Spectra	PCA	LDA
Vicente et al.[9]	2013	*not reported*	Metabolite concentrations	PCA	LDA
Zarinabad et al.[10]	2017	*not reported*	Metabolite concentrations	PCA	LDA, NB, NN, SVM
		not reported	Spectra	PCA	LDA, NB, NN, SVM
Zarinabad et al.[11]	2018	*not reported*	Metabolite concentrations	PCA	SVM
Manias et al.[12]	2018	*not reported*	Metabolite concentrations	*not reported*	*not reported*
Davies et al.[7]	2022	*not reported*	Metabolite concentrations	PCA	LDA
Zhao et al.[13]	2022	*not reported*	Metabolite concentrations	mAUC	kNN, LDA, NB, NN, SVM
Zhao et al.[14]	2024	AWNS	Metabolite concentrations	mAUC	kNN, LDA, NB, NN, SVM
dMRI and ^{1}H-MRS					
Schneider et al.	2007	*not reported*	Metabolite concentrations and raw ADCs	Manual	*not reported*

Abbreviations: ^{1}H-MRS, proton magnetic resonance spectroscopy; ADC, apparent diffusion coefficient; AWNS, adaptive wavelet noise suppression; dMRI, diffusion-weighted magnetic resonance imaging; LDA, linear discriminant analysis; kNN, *k*-nearest neighbours; NB, naïve Bayes; NN, neural network; PCA, principal component analysis; RF, random forest; SVM, support vector machine.
